# Epigenetic Regulation of Airway Epithelium Immune Functions in Asthma

**DOI:** 10.3389/fimmu.2020.01747

**Published:** 2020-08-18

**Authors:** Bilal Alashkar Alhamwe, Sarah Miethe, Elke Pogge von Strandmann, Daniel P. Potaczek, Holger Garn

**Affiliations:** ^1^Institute of Laboratory Medicine, Philipps-University Marburg, Member of the German Center for Lung Research (DZL), Universities of Giessen and Marburg Lung Center, Marburg, Germany; ^2^College of Pharmacy, International University for Science and Technology (IUST), Daraa, Syria; ^3^Center for Tumor Biology and Immunology, Institute of Tumor Immunology, Philipps University Marburg, Marburg, Germany; ^4^Translational Inflammation Research Division & Core Facility for Single Cell Multiomics, Philipps University Marburg, Marburg, Germany; ^5^John Paul II Hospital, Kraków, Poland

**Keywords:** airway, allergy, asthma, epigenetic, epithelium, histone, methylation, microRNA (miRNA)

## Abstract

Asthma is a chronic inflammatory disease of the respiratory tract characterized by recurrent breathing problems resulting from airway obstruction and hyperresponsiveness. Human airway epithelium plays an important role in the initiation and control of the immune responses to different types of environmental factors contributing to asthma pathogenesis. Using pattern recognition receptors airway epithelium senses external stimuli, such as allergens, microbes, or pollutants, and subsequently secretes endogenous danger signaling molecules alarming and activating dendritic cells. Hence, airway epithelial cells not only mediate innate immune responses but also bridge them with adaptive immune responses involving T and B cells that play a crucial role in the pathogenesis of asthma. The effects of environmental factors on the development of asthma are mediated, at least in part, by epigenetic mechanisms. Those comprise classical epigenetics including DNA methylation and histone modifications affecting transcription, as well as microRNAs influencing translation. The common feature of such mechanisms is that they regulate gene expression without affecting the nucleotide sequence of the genomic DNA. Epigenetic mechanisms play a pivotal role in the regulation of different cell populations involved in asthma pathogenesis, with the remarkable example of T cells. Recently, however, there is increasing evidence that epigenetic mechanisms are also crucial for the regulation of airway epithelial cells, especially in the context of epigenetic transfer of environmental effects contributing to asthma pathogenesis. In this review, we summarize the accumulating evidence for this very important aspect of airway epithelial cell pathobiology.

## Introduction

Asthma is a chronic inflammatory disease of the airways, in which airway obstruction and hyperresponsiveness underlie recurrent breathing problems, with symptoms being especially pronounced during disease exacerbations (1, 2). Respiratory tract epithelium plays an important role in asthma by initiating and controlling immune responses to different types of pathogenic environmental factors, including allergens, viruses, pollutants, and others. The biology of the airway epithelium in health and its pathobiology in asthma are regulated by epigenetic mechanisms forming the intercellular homeostatic system responding to internal as well as external changing conditions on the level of transcriptional and posttranscriptional regulation of gene expression (3, 4).

## Airway Epithelium and Asthma

The airway epithelium is the first structure of the body getting into contact with inhaled air with all its containing environmental components. Initially, it was thought to just constitute a mechanical barrier to enable the bidirectional transfer of air to and from the gas-exchanging alveolar structures. Over the last years, it turned out, however, that the airway epithelium in general and the bronchial epithelium as a major part of it in particular represent a much more complex tissue fulfilling a variety of additional functions such as retrograde transport of inhaled particles, establishment of a biochemical barrier system, and initiation and regulation of innate and adaptive immune mechanisms by release of various cytokines and chemokines. By this, it represents an integrative part of the innate immune system, the coordinated activity of which is essential for maintaining the local tissue and even systemic body integrity (5). To exert these diverse functions, the bronchial epithelium is composed of multiple structurally and/or functionally differing cell types, such as ciliated cells (mucociliary transport), goblet cells (mucus secretion), tuft and M cells (luminal signal sampling and antigen presentation), ionocytes (water regulation), and club cells (mucus and surfactant protein production) (6). All these cell types develop from local stem cell precursors, called basal cells (7). It is quite obvious that the continuous development of the different cell types from such precursors, as well as their concerted action under healthy conditions, requires a high level of control and regulation (8). In asthma, the underlying control mechanisms are disturbed by both external (environmental factors such as allergens, pollen, bacteria, viruses) and internal (i.e., cytokines, chemokines, low-molecular-weight mediators produced by innate and adaptive immune cells) influences, resulting in dysregulated activities of the bronchial epithelium (9). This includes hypersecretion of mucus, release of epithelial-derived cytokines called alarmins [e.g., interleukin 25 (IL-25), IL-33, thymic stromal lymphopoietin], chemokines, and antimicrobial peptides, as well as uncontrolled proliferation and differentiation processes, altogether leading to functional [e.g., airway hyperresponsiveness(AHR)] and structural (e.g., airway remodeling) changes that represent characteristic features of asthma pathology (10). Not unexpectedly, because of the close relation to environmental influences and their changes, epigenetic regulation processes are crucially involved in the appropriate development, maintenance, and functionality of the different components of the airway epithelium (11). Chronic inflammatory processes such as in asthma are expected to interfere with these well-balanced epigenetic mechanisms in the epithelium of the airways. This may happen at the level of the aforementioned finally differentiated cell types and by changing related gene expression patterns that influence their functional behavior. It is also conceivable that epigenetic changes occur already at the level of the basal cells, which would then be inherited to all kinds of cells developing from the affected precursors with multiple functional consequences (12). It needs to be considered that these mechanisms may either lead to further perpetuation of the disease process or, alternatively, represent repair activities initiated to get the complex system back to steady state, that is, healthy conditions.

## Epigenetic Mechanisms

**Figure 1:**
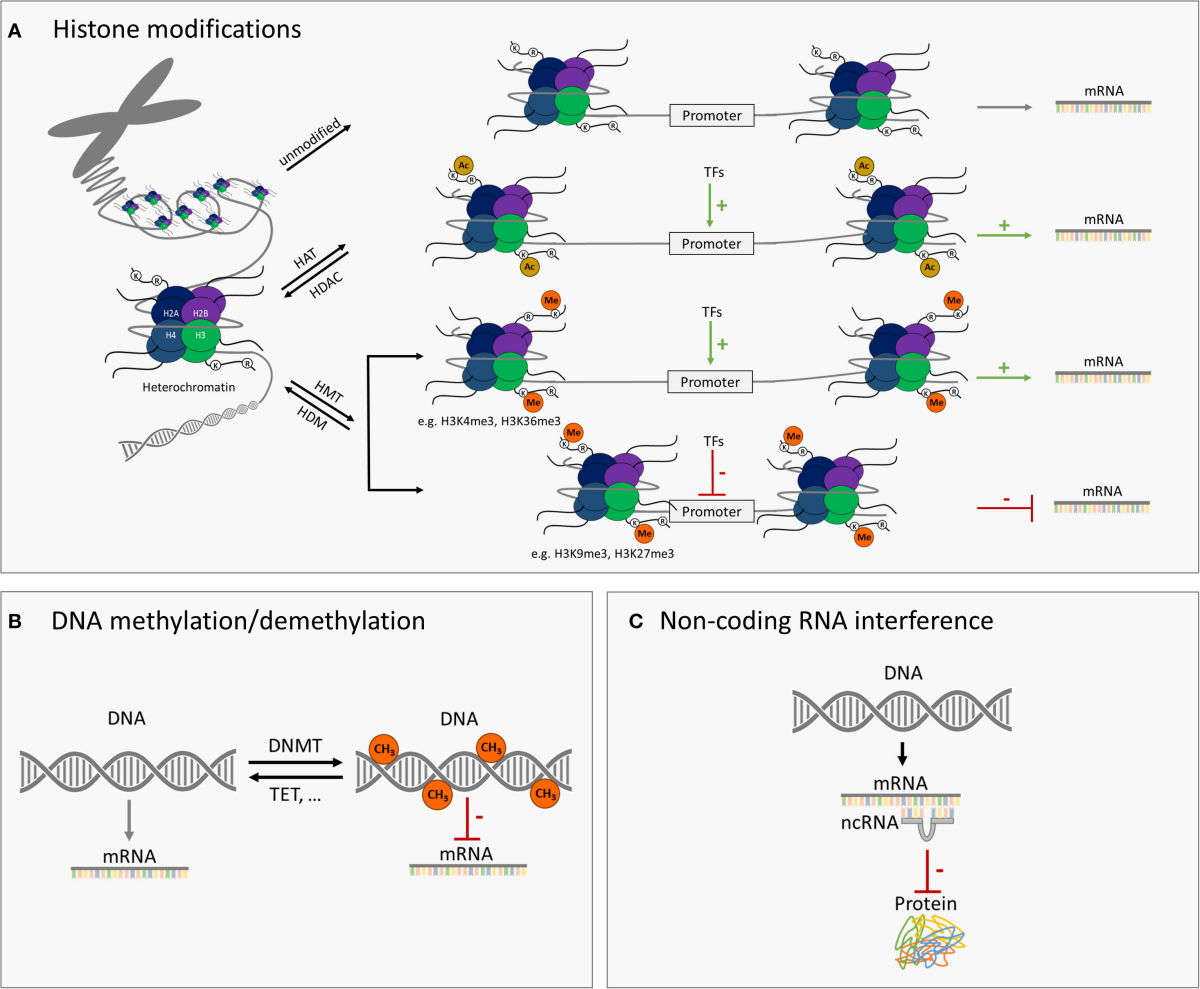
Schematic illustration of major epigenetic modifications. **(A)** Modification of histones such as histone acetylation/deacetylation via histone acetyltransferases (HATs)/histone deacetylases (HDAC) and methylation/demethylation via histone methyltransferases (HMT)/histone demethylases (HDM) can either activate or repress the target gene transcription. Histone acetylation is typically associated with higher expression of the gene. Histone methylation can be related to either higher or lower transcriptional activity, depending on the amino acid residue modified and the number of methyl groups added. **(B)** DNA methylation or demethylation of genomic DNA through DNA methyltransferases (DNMT) or ten-eleven translocation (TET) enzymes and others, respectively. Higher level of DNA methylation is typically associated with lower transcriptional activity of the respective gene. **(C)** MicroRNAs (miRNAs) and further small non-coding RNAs can interfere with gene expression through base pairing with messenger RNAs and thus inhibiting their translation into the encoded protein.

Epigenetics comprises molecular mechanisms of inheritable but reversible phenotypic changes that lead to modified gene expression without alterations at the level of the DNA sequence (13). In the human genome, 80% of the DNA is packed into nucleosomes, and the rest forms linkers between nucleosomes. The nucleosomes are further packed into dense three-dimensional structures called chromosomes (14). The core components of the nucleosome are histone proteins, which are accessible to different types of posttranslational modifications (PTMs), including acetylation, methylation, phosphorylation, sumoylation, and ubiquitination. Posttranslational modifications, especially if occurring at important regulatory genomic regions such as enhancers or promoters, are able to change the accessibility of the DNA to the transcriptional machinery, which is associated with active, poised, or silenced status of transcriptional activity. For example, histone acetylations, the changes introduced by histone acetyltransferases (HATs) and removed by histone deacetylases (HDACs), are usually associated with transcriptional activation of the gene (15, 16). DNA methylation, in which a methyl group is enzymatically added to the cytosine ring of DNA, is another type of the epigenetic modification. While the methylation reaction is catalyzed by DNA methyltransferases, ten-eleven translocation (TET) methylcytosine dioxygenase family proteins mediate DNA demethylation. DNA methylation is typically associated with gene repression (3, 17). In addition to the classical epigenetic modifications mentioned above, different types of the non-coding RNAs such as microRNAs (miRNAs) and others, for instance, piwi-interacting RNAs or small nucleolar RNAs, are involved in the epigenetic regulation of gene expression. Briefly, miRNAs exert their silencing effects through the binding to the mature mRNA molecules in the cytosol that leads to mRNA degradation or reduction in the translational efficiency of the ribosomes (18, 19) (Figure 1).

## DNA Methylation

**Figure 2:**
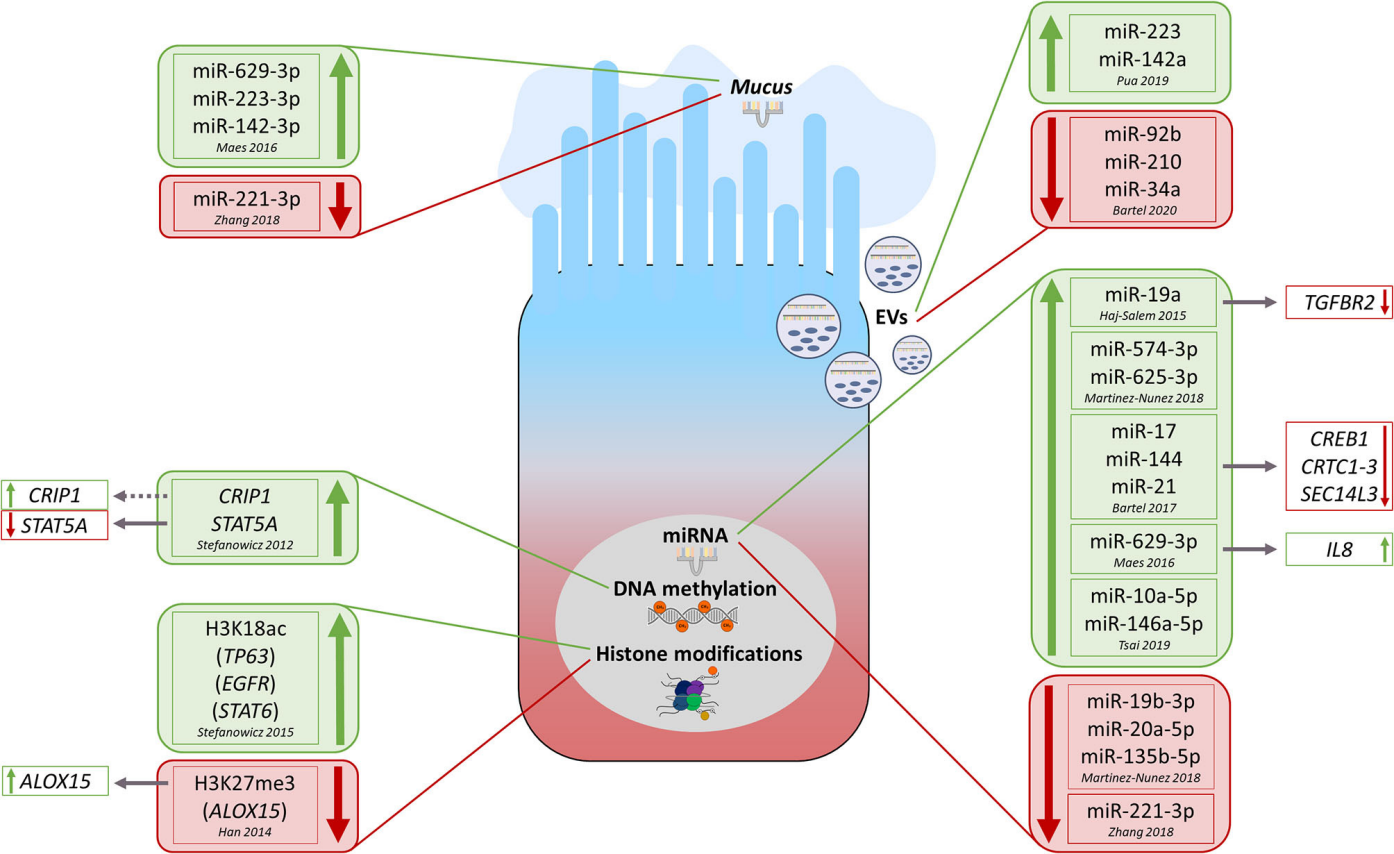
Overview of currently known key epigenetic modifications observed in lower airway epithelial cells from asthma/allergic airway inflammation conditions and—if known—associated functional consequences. The green color always indicates upregulation of the respective modification in asthmatics vs. healthy while red color identifies opposite regulation. EVs, extracellular vesicles; miRNA, microRNA; H3K18ac, histone H3K18 acetylation; H3K27me3, histone H3K27me3 trimethylation.

DNA methylation is probably the best studied epigenetic modification in general but also in relation to asthma. Although studies conducted so far on the involvement of DNA methylation in asthma have mostly used already available DNA samples and/or DNA extracted from easily available tissues, also lower airway epithelial cells (AECs) have been investigated (Figure 2). Stefanowicz et al. (20) performed a comparative DNA methylation analysis of 807 genes in bronchial AECs and peripheral blood mononuclear cells (PBMCs) obtained from atopics, atopic asthmatics, non-atopic asthmatics, or healthy controls. They identified signature sets of CpG sites differentially methylated between AECs and PBMCs, which were either independent of the disease phenotype or specific to healthy controls, atopics, or asthmatics. Although no differences in the DNA methylation status were found between disease phenotypes in PBMCs, they were observed between asthmatics and atopics in AECs (20). Kim et al. (21) comparatively analyzed genome-wide DNA methylation levels in bronchial mucosa tissues obtained from atopic and non-atopic asthmatics and healthy controls. Although the methylation levels were similar between asthmatics and controls, a set of loci has been identified with significant differences in DNA methylation between atopic and non-atopic asthmatics (21). Clifford et al. (22) investigated in turn the effects of experimental respiratory tract exposure to allergen, diesel exhaust, or both as a coexposure, always observing only minimal resulting changes in the bronchial epithelial DNA methylome of the participating individuals. They found, however, that if any of the two insults occurs in advance of the other (crossover exposure with a 4-week interval), the initial one primes the bronchial epithelial DNA methylome for the second, resulting in cumulative epigenetic changes with potential biological relevance (22).

In most cases, however, DNA methylation studies conducted in airway tissue in the context of asthma have not been performed in bronchial or lung ACEs but rather in nasal epithelial cells (NECs) due to easier accessibility (23–28). Cardenas et al. (23) conducted an epigenome-wide study on DNA methylation using nasal swabs collected in a large group of early teenagers deriving from a birth cohort. They identified multiple DNA methylation loci associated with asthma, allergies, and related clinical or laboratory parameters (23). In another cohort of adolescents, Forno et al. (24) performed in turn an epigenome-wide analysis of DNA methylation in nasal epithelium. The major findings of this study, replicated in two independent cohorts, comprised the identification of specific DNA methylation profiles associated with atopy and atopic asthma and a nasal methylation panel that could classify children by atopy or atopic asthma (24). Reese et al. (25) sought to identify differential DNA methylation related to pediatric asthma in blood from newborns and school-aged children. Interestingly, they were able to replicate in eosinophils or nasal respiratory epithelium most of the asthma-related differential methylation signatures initially detected in blood (25). Brugha et al. (29) comparatively analyzed DNA methylation in airway and surrogate tissues. They found that the methylation profile in nasal epithelium was most representative of that in the airway epithelium, whereas the profile in buccal cells was moderately and that in blood was least similar (29). In our view, these results clearly suggest that DNA methylation studies performed in the context of asthma as an airway disease should preferentially be conducted using AECs or NECs. Although beyond the scope of this review, we would like to mention that, in our view, sorted specific white blood cell populations would be highly valuable to study systemic adaptive immunity DNA methylation patterns underlying asthma. However, how well those signatures correspond to local lung DNA methylation patterns would need to be assessed in separate studies. Back to NECs, Xiao et al. (27) showed that nasal DNA methylation at the promoter of the vanin 1 gene (*VNN1*) might be a clinically useful biomarker of corticosteroid treatment response in asthmatic children. Another study from the same group demonstrated in turn that DNA methylation at the TET methylcytosine dioxygenase 1 gene (*TET1*) contributes to traffic-related air pollution and asthma (26).

Finally, allelic differences in DNA methylation and thus gene expression in AECs can mediate the effects of certain genetic variants known to be associated with susceptibility to childhood asthma, such as those in chromosome 17q21 (30, 31).

## Histone Modifications

In addition to DNA methylation, also histone modifications participate in epithelial (patho-) mechanisms related to asthma (Figure 2). Stefanowicz et al. (32) compared global and gene-specific alveolar epithelial cells histone acetylation and methylation status between asthmatics and healthy subjects. Generally, they observed higher global H3K18ac and H3K9me3 levels in asthmatic subjects. In more detail, they found in asthmatics a higher association of H3K18ac (but not H3K9me3) around the transcription start sites of *TP63* (tumor protein p63, ΔNp63 isoform), *EGFR* (epidermal growth factor receptor), and *STAT6* (signal transducer and activator of transcription 6) genes. Finally, they detected a non-significant increase in protein expression of those three genes in AECs treated with trichostatin A, an HDAC inhibitor (HDACi) (32). In another work, the same group comparatively analyzed the expression of 82 epigenetic modifying enzymes in AECs and bronchial fibroblasts obtained from asthmatics and healthy controls (33). Thirty-nine enzymes were differentially expressed between AECs and bronchial fibroblasts, 24 of which passed the correction for multiple testing. Six histone modifiers turned out to be differentially expressed in AECs between asthmatics and non-asthmatics, however, mostly not significantly when corrected for multiple testing (33).

Beneficial effects of HDACi have been observed in murine models of allergic airway inflammation (AAI) mimicking features of human allergic asthma (34, 35). Application of HDACi in an ovalbumin (OVA)–based model reduced airway inflammation, remodeling, and AHR. In addition, HDACi treatment was associated with lower expression of transforming growth factor β1 (TGF-β1) in AECs and diminished synthesis of contractile proteins by airway smooth muscle cells (34). HDACi treatment in mice subjected to a house dust mite (HDM)–based model was in turn able to prevent them from developing AHR and AAI. Moreover, HDACi restored the integrity of the *ex vivo*–cultured NECs isolated from AR patients (35). Significantly lower H3K27me3 levels at the promoter of the arachidonate 15-lipoxygenase (ALOX15) gene (*ALOX15*) were observed in human lung epithelial A549 cells after the treatment with IL-4, which coincided with higher *ALOX15* mRNA levels (36).

Targeting histone modification—related mechanisms turned out to be effective also in a cockroach allergen extract–induced mouse model of mixed granulocytic (eosinophilic and neutrophilic), T_H_2/T_H_17-driven asthma (37). Specifically, whereas a bromo- and extraterminal (BET) inhibitor was already alone able to abolish T_H_17-driven neutrophilic inflammation, in combination with dexamethasone it completely blocked both T_H_2- and T_H_17-driven immune responses in the lung, which was associated with reductions in lung eosinophilia and neutrophilia, and mucin secretion. Furthermore, BET inhibition improved cockroach allergen extract– or IL-17A–induced increase in markers of glucocorticoid insensitivity [i.e., decrease in HDAC2 expression (38)] in murine or human AECs, respectively (37). In another study, *Hdac2*^+/−^ mice subjected to an HDM-induced AAI model demonstrated stronger inflammatory infiltration as well as higher expression of type 2 cytokines and IL-17A in the lung tissue compared to wild-type animals. Additional IL-17A depletion was able to reverse these HDAC2 impairment-induced effects (39). In turn, HDM and IL-17A synergistically reduced HDAC2 expression in human bronchial epithelial cells (BECs) *in vitro*. Besides, silencing the HDAC2-encoding gene further enhanced HDM- and/or IL-17A–induced inflammatory cytokines in human BECs, whereas HDAC2 overexpression or knockdown of the gene encoding IL-17A was able to reduce the release of such inflammatory cytokines (39). Taken together, original findings by Zijlstra et al. (38), who first discovered IL-17A–induced steroid resistance mediated by a reduction of HDAC2 activity, have thus been corroborated and expanded.

## Microrna

Several recent studies have highlighted the importance of miRNAs in the regulation of epithelial pathobiology in asthma (Figure 2). Bartel et al. (40) combined different approaches such as *in vivo* studies in mice with OVA- or HDM-induced AAI, *ex vivo*/*in vitro* experiments including luciferase reporter assay and stimulation–expression analyses, miRNA/mRNA microarrays, and *in silico* approaches. This composed strategy enabled the authors to identify the transcription factor cAMP-responsive element binding protein (*Creb1*) and its transcriptional coactivators (*Crtc1*-*3*) as targets for miR-17, miR-144, and miR-21, all three deregulated in lungs of mice with AAI. Moreover, they observed downregulation of Sec14-like 3 (*Sec14l3*), a putative target of Creb1, in both AAI models and in primary normal human BECs upon IL-13 treatment suggesting that miRNA-regulated Crtc1-3 and Sec14l3 play a role in early epithelial responses to type 2 stimuli (40). Microarray analysis of miRNA expression in bronchoscopy-isolated human BECs showed in turn an upregulation of miR-19a in samples obtained from severe asthmatic subjects compared to those from mild asthmatics and healthy controls (41). Furthermore, luciferase reporter assay- and Western blot–based functional studies demonstrated miR-19a to enhance proliferation of BECs in severe asthma through targeting TGF-β receptor 2 gene (*TGFBR2*) mRNA (41). Using subcellular fractionation and RNA sequencing (Frac-seq) in human primary BECs from healthy controls and severe asthmatics, Martinez-Nunez et al. (42) assessed paired genome-wide expression of miRNAs along with cytoplasmic (total) and polyribosome-bound (translational) mRNA levels. They identified a hub of six dysregulated miRNAs, displaying preference for polyribosome-bound mRNAs, which accounted for ~90% of whole miRNA targeting. Interestingly, transfection of such miRNAs into BECs obtained from healthy subjects turned them into cells mimicking features of those obtained from severe asthmatics (42).

Recently, extracellular vesicles (EVs) transferring miRNAs between cells have been identified as a novel mechanism of intercellular communication (3, 43, 44). Of note, the composition of the extracellular miRNA pool in the lung of mice was very similar to that of the airway epithelium, and ~80% of the detected EVs were of epithelial origin (45). However, following the induction of AAI, the presence of miRNAs preferentially expressed by immune cells, such as miR-223 and miR-142a, and hematopoietic cell–derived EVs increased also substantially, indicating an importance of the extracellular miRNA pool for the development of local allergic inflammatory processes (45). Gupta et al. (46) focused on EVs secreted by two kinds of human airway cell cultures, that is, primary tracheobronchial cells and a cultured AEC line (Calu-3). Their data suggest that cellular information can be transferred between AECs via miRNA-containing EVs, which may thereby contribute to epithelial biology and remodeling (46). Another study profiled the expression of miRNAs in EVs secreted from the apical and basal sides by normal human BECs treated with IL-13 in order to induce an asthma-like response (47). Significant candidates were then confirmed in EVs isolated from nasal lavages obtained from children with mild to moderate or severe asthma and healthy control subjects. Interestingly, levels of miR-92b, miR-210, and miR-34a turned out to correlate with lung function measures (47).

Two studies investigated miRNAs in asthmatic sputum (48, 49). In two independent cohorts, Maes et al. (48) found a significant upregulation of miR-629-3p, miR-223-3p, and miR-142-3p in sputum of severe asthmatics compared to healthy controls, with the highest levels in patients with neutrophilic asthma. Of those three miRNAs associated with sputum neutrophilia and airway obstruction, miR-629-3p was expressed in BECs. Interestingly, transfection of human BECs with a miR-629-3p mimic induced expression of IL-8, the sputum levels of which were significantly increased and positively correlated with sputum neutrophilia in severe asthmatics (48). Zhang et al. (49) found in turn that epithelial, sputum, and plasma miR-221-3p levels were significantly lower in asthmatics compared to healthy controls. In addition, levels of epithelial and sputum miR-221-3p inversely correlated with airway eosinophilia (49).

Finally, Tsai et al. (50) sought to find the common miRNA-related effects in BECs obtained from subjects with asthma and chronic obstructive pulmonary disease (COPD). First detected with next-generation sequencing, the upregulation of miR-10a-5p and miR-146a-5p in BECs obtained from both asthma and COPD patients was subsequently confirmed by quantitative polymerase chain reaction. Moreover, compared to healthy controls, also serum miR-146a-5p levels were higher in asthma and COPD subjects (50). Further research will establish whether miRNAs mediating intercellular communication can be used for clinical applications as biomarkers or therapeutic targets.

## Special Aspects

Airborne viruses, for instance, human rhinoviruses (HRVs), stimulate asthma exacerbations. In addition, repeated early life infections with such viruses can lead to the development of a persistent asthma phenotype, especially in children with atopic susceptibility (3, 51). Interestingly, some studies suggest that the effects of respiratory viral infections are at least partly mediated by epigenetic changes in airway epithelial cells. It has been demonstrated that *ex vivo* experimental HRV infection of NECs obtained from asthmatic children significantly changes patterns of DNA methylation and mRNA expression (52). Moreover, HRV infection in young children has been associated with changes in the airway secretory miRNome, characterized by a highly specific additional appearance of miR-155 in nasal secretion EVs (53). In turn, BECs obtained from asthmatics have been shown to be characterized by dysregulated miR-22 expression after experimental *ex vivo* infection with influenza A virus (IAV) (54). Other epigenetic modifications, specifically histone methylations, also seem to be involved in the regulation of epithelial antiviral responses (55).

Dysregulated epithelial–mesenchymal transition (EMT) is the process driven mostly by TGF-β1, which strongly contributes to the establishment of the persistent asthma phenotype, that is, to disease chronification (56). Epigenetic mechanisms seem to play an important role in EMT. It has been demonstrated in mouse models mimicking allergic asthma that miR-448-5p can inhibit TGF-β1–induced EMT and pulmonary fibrosis (57). Applying an epigenome-wide approach in a human study, McErlean et al. (58) identified in turn multiple loci showing differential H3K27ac enrichment in asthma, which clustered at genes associated with type 2–driven asthma and EMT.

## Conclusions and Perspectives

Epigenetic mechanisms play a very important role in the epithelial pathobiology of asthma. While histone modifications seem to be especially interesting as possible therapeutic targets, DNA methylation and miRNAs, also from the easily accessible nasal epithelium, show a substantial diagnostic potential. Although the data gathered by now (for overview, see also Supplementary Table 1) already strongly suggest a usefulness of epigenetics in the asthma management, further studies, especially those considering the complex interplay of different epigenetic mechanisms and those focusing on a single-cell type or investigations on the single cell level, are needed.

## Author Contributions

BA: draft writing and figure drafts. SM: draft writing and final figures. ES: editing and reviewing. DP: conceptualization, draft writing, and reviewing. HG: conceptualization, coordination, draft writing, and reviewing. All authors contributed to the article and approved the submitted version.

## Conflict of Interest

The authors declare that the research was conducted in the absence of any commercial or financial relationships that could be construed as a potential conflict of interest.
