# Supplementary material for: Epigenetic Regulation of Airway Epithelium Immune Functions in Asthma
Source: Front Immunol. 2020 Aug 18;11:1747. doi: 10.3389/fimmu.2020.01747 (PMC7461869; doi:10.3389/fimmu.2020.01747)
Supplement: Supplementary file 1 [file Data_Sheet_1.PDF]

Supplementary Table 1. Selected studies showing the involvement of epigenetic changes in human airway epithelial cells in asthma pathogenesis.

**Epigenetic targets**	**Biomaterials**	**Key results**	**References**
DNA methylation;<br>807 genes	Bronchial epithelial cells obtained from atopics, atopic asthmatics, non-atopic asthmatics, or healthy controls	A set of differentially methylated sites identified between cells deriving from asthmatics and atopics, including CpGs in *STAT5A* and *CRIP1*	Stefanowicz at al., 2012
DNA methylation;<br>epigenome-wide	Bronchial mucosa tissues obtained from atopic and non-atopic asthmatics, and healthy controls	No evident differences in methylation between asthmatics and controls; a set of loci showing significant differences between atopic and non-atopic asthmatics identified	Kim et al., 2013
DNA methylation;<br>epigenome-wide	Nasal swabs collected in a large group of early teenagers deriving from a birth cohort	Multiple loci associated with asthma, allergies, and related clinical or laboratory parameters	Cardenas et al., 2019
DNA methylation;<br>epigenome-wide	Nasal epithelium obtained from a cohort of adolescents	Specific methylation profiles associated with atopy and atopic asthma, and a nasal methylation panel classifying children by atopy or atopic asthma identified	Forno et al., 2019
Histone acetylation and methylation;<br>global and gene-specific	Alveolar epithelial cells obtained from asthmatic and healthy subjects	Higher global H3K18ac and H3K9me3 levels in asthmatics; a higher association of H3K18ac (but not H3K9me3) around the transcription start sites of *TP63* (ΔNp63 isoform), *EGFR*, and *STAT6* in asthmatics	Stefanowicz at al., 2015
Histone modifications;<br>expression of key enzymes involved in epigenetic mechanisms	Airway epithelial cells from asthmatics and non-asthmatics	Six histone modifiers differentially expressed between asthmatics and non-asthmatics (although mostly not significantly after correction for multiple testing)	Stefanowicz at al., 2017
Histone acetylation;<br>effects of HDACi treatment	Nasal epithelial cells from allergic rhinitis patients	Integrity of the *ex vivo*-cultured cells restored after HDACi treatment	Steelant et al., 2019
Histone methylation	Human lung epithelial A549 cells	Significantly lower H3K27me3 levels at the *ALOX15* promoter after treatment with IL-4	Han et al., 2014
Histone acetylation;<br>expression of HDAC2 and related effects	Human bronchial HBE135-E6E7 cells	Synergistic reduction of HDAC2 expression after HDM and IL-17A treatment; enhancement of HDM- and/or IL-17A-induced inflammatory cytokines after HDAC2 silencing; reduction in the release of inflammatory cytokines following HDAC2 overexpression or IL-17A knock-down	Lai et al., 2019

Epigenetic targets	Biomaterials	Key results	References
miRNA;<br>377 targets	Bronchoscopy-isolated human bronchial epithelial cells from severe or mild asthmatics and healthy controls	Upregulation of miR-19a in samples obtained from severe asthmatics compared to mild asthmatics or healthy controls; enhancement of proliferation of bronchial epithelial cells obtained from severe asthmatics through downregulation of *TGFBR2* expression by miR-19a	Haj-Salem et al., 2015
miRNA;<br>genome-wide with subcellular fractionation	Bronchoscopy-isolated human bronchial epithelial cells from severe asthmatics and healthy controls	A hub of six dysregulated miRNAs, displaying preference for polyribosome-bound mRNAs, accounting for about 90% of whole miRNA targeting; bronchial epithelial cells from healthy subjects transfected with those miRNAs mimicking features of those obtained from severe asthmatics	Martinez-Nunez et al., 2018
miRNA;<br>384 targets	EVs secreted by normal human bronchial epithelial cells treated with IL-13; EVs isolated from nasal lavages obtained from children with mild-to-moderate or severe asthma and healthy controls	Significant differentially expressed miRNAs after IL-13 stimulation confirmed in EVs isolated from nasal lavages from patients and controls; correlation of miR-92b, miR-210, or miR-34a levels with lung function measures	Bartel et al., 2020
miRNA;<br>755 targets	Sputum from asthmatics (severe or mild-to-moderate; eosinophilic or neutrophilic asthma) and healthy controls	Significant upregulation of miR-629-3p (expressed in bronchial epithelial cells), miR-223-3p, and miR-142-3p in sputum of severe asthmatics compared to healthy controls, with the highest levels in patients with neutrophilic asthma; induction of IL8 expression (sputum levels of which associated with severe asthma and positively correlating with sputum neutrophilia) in human bronchial epithelial cells transfected with a miR-629-3p mimic	Maes et al., 2016
miRNA;<br>target-specific	Bronchoscopic airway epithelial brushings and sputum from asthmatics and healthy controls	Levels of miR-221-3p significantly lower in asthmatics compared to healthy controls	Zhang et al., 2018a
miRNA;<br>genome-wide	Bronchial epithelial cells from patients with asthma, COPD, or normal controls	Upregulation of miR-10a-5p and miR-146a-5p in cells obtained from subjects with asthma or COPD	Tsai et al., 2019

HDACi, histone deacetylase (HDAC) inhibitor; IL, interleukin; HDM, house dust mite; EV, extracellular vesicle; COPD, chronic obstructive pulmonary disease
